# Halide-Assisted Synthesis of V-WSe_2_

**DOI:** 10.3390/ma18235360

**Published:** 2025-11-28

**Authors:** Yanhui Jiao, Xiaoqian Wang, Zisheng Tang, Manrui Liu, Chengqi Liu, Qi Zhang, Yong Liu

**Affiliations:** State Key Laboratory of Advanced Technology for Materials Synthesis and Processing, International School of Materials Science and Engineering (ISMSE), Wuhan University of Technology, Wuhan 430070, China; j1769877205@163.com (Y.J.); 303568@whut.edu.cn (X.W.); tangzs3076@163.com (Z.T.); liumanr14@163.com (M.L.); liuchengqi42@163.com (C.L.); zq13307239180@163.com (Q.Z.)

**Keywords:** TMDCs, synthesis, two-dimensional materials

## Abstract

Over the past few years, two-dimensional transition metal dichalcogenides (TMDCs) have garnered substantial attention in the field of two-dimensional materials research, owing to their exceptional physicochemical properties. Notably, V-WSe_2_ distinguishes itself by reducing the Schottky barrier at the interface between the material and metal electrodes, thus exhibiting remarkable potential for applications in optoelectronic devices. Our work explores the synthesis of monolayer V-WSe_2_ through halide-assisted atmospheric-pressure chemical vapor deposition (APCVD), with an emphasis on the effects of various halide types on the growth mechanism. In addition, we investigate the impact of vanadium (V) content on the performance of WSe_2_. Comprehensive optical and structural characterizations of the synthesized material were systematically performed. The findings indicate that incorporating halide salts effectively reduces the volatilization temperature of tungsten trioxide (WO_3_), thereby markedly enhancing reaction controllability and material crystallinity. Among the tested halide salts, KCl, NaCl, and KI, KI demonstrated the capability to achieve the lowest growth temperature. Varying the V content in the V-WSe_2_ structure significantly influences the optical properties, with higher vanadium concentrations reducing the material’s optical bandgap and Raman frequency. This study highlights the critical role of halides and vanadium content in the material growth process, providing valuable insights for the controlled synthesis of two-dimensional TMDC materials and how varying vanadium concentrations also affect the material’s performance.

## 1. Introduction

Since the isolation of monolayer graphene using adhesive tape in 2004 [1], two-dimensional (2D) materials have garnered significant attention, ushering in a new wave of research. Compared with bulk materials, 2D materials, comprising a single or a few atomic layers, exhibit a range of unique and compelling properties [2,3,4,5,6,7]. TMDCs, characterized by the general formula MX_2_ (where M denotes a transition metal and X represents a chalcogen element), are among the most representative 2D materials. The physicochemical properties of these materials are governed by the specific combination of transition metals and chalcogen elements [8,9]. For instance, MoS_2_ and WS_2_ demonstrate a direct bandgap in their monolayer form, making them highly attractive for optoelectronic applications, including photodetectors, light-emitting diodes (LEDs), and transistors, whereas bulk counterparts typically exhibit an indirect bandgap. Two-dimensional TMDCs have emerged as fundamental materials for the advancement of next-generation electronic devices, owing to their unique electronic properties, particularly in monolayer form [10,11,12]. Owing to their atomic-scale thickness, dangling-bond-free surfaces, excellent flexibility, and semiconducting characteristics, monolayer TMDCs have attracted widespread attention for their potential applications in nanoscale electronics [13,14], high-efficiency photodetectors [15,16], and wearable devices [17,18]. The integration of 2D TMDC materials into silicon-based integrated circuits facilitates the extension of Moore’s Law while simultaneously achieving improved performance [19,20]. Furthermore, 2D TMDCs have been extensively utilized in emerging technologies such as optoelectronics, spintronics, and quantum computing [21,22]. Tungsten diselenide (WSe_2_) is widely regarded as one of the most promising semiconductors, owing to its notable bandgap, exceptional air stability, and outstanding switching ratio, thereby garnering significant attention [23,24]. Fermi-level pinning at the metal/semiconductor interface gives rise to a Schottky contact, which remains a major obstacle, significantly affecting the transport characteristics of WSe_2_-based field-effect transistors (FETs) [25,26,27]. To address this issue, modulating the WSe_2_ work function by controlling the carrier concentration is crucial for reducing the Schottky barrier (SB). For example, vanadium doping in WSe_2_ enhances the energy band position of WSe_2_, strengthens the source field to lower the Schottky barrier, improves hole tunnelling, and reduces the contact resistance. This enhancement optimizes the performance of the field-effect transistor by promoting hole tunneling and increasing carrier mobility [28]. The high carrier mobility and strong light–matter interaction of V-WSe_2_ support ultrafast photodetection in the visible to near-infrared spectrum. These properties are critical for imaging systems, optical communications, and AI-driven sensor networks. Doping has been demonstrated to be an effective means of tuning the work function, bandgap, and various other properties of TMDCs, facilitating the emergence of novel physical phenomena and promising applications.

In the past decade, a wide array of doping strategies has been developed to enhance the performance of WSe_2_. These strategies encompass the physical adsorption of electrostatic doping [29], substitutional doping [30], aromatic molecules [31], the introduction of various adsorbate atoms [32,33], laser-assisted doping [34,35,36], and plasma doping [37,38]. Among these, substitutional doping is regarded as the most stable and effective technique, wherein intentionally introduced impurity atoms replace metal or chalcogen atoms, forming covalent bonds with the host lattice, resulting in more robust structures and enhanced material properties. Furthermore, pioneering research has employed chemical vapor transport (CVT) growth to synthesize bulk MoS_2_ and WSe_2_ crystals doped with Nb, Re, and V, followed by mechanical exfoliation to obtain monolayers or few-layer flakes. This method enables precise control over doping concentration without compromising the intrinsic properties of the material, thereby further optimizing semiconductor performance [28,39,40]. These groundbreaking studies have established a robust foundation for the advancement of doping technologies and the application of materials such as WSe_2_ in electronic devices. Among various synthesis methods, chemical vapor deposition (CVD) has emerged as a prominent method, due to its ability to synthesize TMDC materials under ambient pressure with highly controllable growth, excellent reproducibility, and stability [41,42,43,44]. Consequently, the majority of contemporary research focuses on the CVD synthesis of TMDCs [45]. During the CVD synthesis process, the reaction parameters, including carrier gas flow rate, precursor–substrate distance, precursor type, and growth temperature, can be adjusted to tailor the properties of the synthesized materials. However, the precise and controlled synthesis of vanadium-doped WSe_2_ (V-WSe_2_) using CVD remains largely unexplored. In conventional methods, the high volatilization temperature of tungsten trioxide (WO_3_), which exceeds 1700 °C, limits the complete reaction between selenium powder and tungsten oxide during the preparation of V-WSe_2_. Therefore, optimizing the volatilization process of WO_3_ is critical for the fabrication of large-area, high-quality, 2D vanadium-doped WSe_2_.

To address this challenge, this study employs a halide-assisted atmospheric-pressure CVD method to successfully synthesize large-area monolayer V-WSe_2_ with varying V concentrations (1%, 2%, 5%, 10%, 15%). During heating, halides react with WO_3_ to form low-melting-point compounds such as WOX_4_ or WO_2_X_2_ [46] (X = Cl or I), which effectively promote the volatilization of tungsten oxide and enhance its solubility and transportability. By introducing halide salts (e.g., KCl, NaCl, KI) to assist in the volatilization of WO_3_, the study investigates the effects of different halides on the growth process of V-WSe_2_. Optical microscopy was used to analyze the morphology of the V-WSe_2_ samples synthesized with various halide additives, revealing the critical role of halides and vanadium content in the growth process. The synthesized materials were further subjected to a series of characterizations to evaluate their properties.

## 2. Materials and Methods

### 2.1. Materials

Precursor: Tungsten trioxide powder (WO_3_, Aladdin, 99.99%, Shanghai, China), vanadium oxide (V_2_O_5_, Macklin, 99.99%, Shanghai, China) and selenium powder (Se, Aladdin, 99%, Shanghai, China); Halide: KI (Aladdin, 99%, Shanghai, China), dicalcium phosphate (KCl, Sinopharm Chemical Reagent, 99.9%, Shanghai, China), sodium chloride (NaCl, Sinopharm Chemical Reagent, 99.9%, Shanghai, China); Organic reagent: Anhydrous ethanol (C_2_H_6_O, Sinopharm Chemical Reagent, AR, Shanghai, China) and acetone (C_3_H_6_O, Sinopharm Chemical Reagent, AR, Shanghai, China); Gas: Argon (Ar gas, Wuhan Newradar Special Gas Co., Ltd., 99.999%, Wuhan, China) and hydrogen–argon mixture gas (Ar/H_2_ gas, Wuhan Newradar Special Gas Co., Ltd., 99.999%, Wuhan, China); Substrate: Si/SiO_2_ (Hefei Kejing Materials Technology Co., Ltd., Hefei, China).

### 2.2. Growth Preparation and Processes

In the halide-assisted atmospheric-pressure chemical vapor deposition (APCVD) synthesis of V-doped WSe_2_, the types and quantities of the precursors are outlined as follows: Tungsten source: 500 mg of WO_3_ powder, thoroughly mixed with 25 mg of halide (KCl/KI/NaCl). Selenium source: 200 mg of Se powder. Vanadium source: V_2_O_5_ powder was precisely weighed according to the target doping concentrations and co-located with the WO_3_/halide mixture. The amounts used were as follows: 1% V-WSe_2_: 5 mg V_2_O_5_; 2% V-WSe_2_: 10 mg V_2_O_5_; 5% V-WSe_2_: 25 mg V_2_O_5_; 10% V-WSe_2_: 50 mg V_2_O_5_; 15% V-WSe_2_: 75 mg V_2_O_5_. The WO_3_ powder was placed at the center of the heating zone in a single-zone tube furnace, with V_2_O_5_ and WO_3_ powders loaded into the same ceramic boat, where V_2_O_5_ was positioned downstream of WO_3_. Selenium (Se) powder was placed upstream, 10 cm away from the WO_3_ powder, and a Si/SiO_2_ substrate was positioned between the V_2_O_5_ and WO_3_ powders. The tube furnace was heated to a temperature range of 800–900 °C within 60 min and maintained at the target temperature for 10 min. Prior to heating, a flow of argon (300 sccm) was introduced to purge the quartz tube and eliminate residual air. During the heating process, the argon flow rate was adjusted to 100 sccm. Due to the relatively weak reductive properties of selenium powder, when the furnace reached the growth temperature, the argon flow was replaced with an Ar/H_2_ gas mixture (10% H_2_) to facilitate the reduction of tungsten oxide by selenium. After the reaction was complete, the Ar/H_2_ flow was terminated, and the argon flow was restored to 100 sccm to prevent contamination from external impurities during the cooling process. Once the furnace had cooled to room temperature, the substrate was retrieved, and the sample was subjected to a series of characterizations.

### 2.3. Characterization

Optical microscope (OM): Sunwoo RX50M microscope (Yuyao, China). The surface morphology and macroscopic features of the samples were analyzed using the Sunwoo RX50M metallurgical microscope.

Scanning Electron Microcopy (SEM): JEM-1400Plus (JEOL, Tokyo, Japan) at an acceleration voltage of 5 kV was employed to analyze the films.

X-ray Photoelectron Spectroscopy (XPS): High-resolution XPS data were obtained by a Thermo Scientific spectrometer ( Waltham, MA, USA) with a monochromatic Al Kα X-ray source (hν = 1486.7 eV) in a high-vacuum environment, which provides a focused beam (spot size ~500 μm) with energy resolution <0.5 eV, ensuring accurate determination of elemental oxidation states and bonding environments.

Photoluminescence (PL) and Raman: PL and Raman spectra of the V-WSe_2_ samples were obtained by a Horiba Raman microscope with a 325 nm laser and 532 nm laser, respectively. All spectra were baseline-corrected and normalized to substrate signals.

Atomic Force Microscopy (AFM): Bruker Dimension FastScan AFM (Billerica, MA, USA) in knockdown mode (scan rate: 1 Hz, resolution: 512 × 512 pixels).

Transmission Electron Microscopy (TEM): As-grown V-WSe_2_ films were lifted from SiO_2_/Si substrates and transferred to Cu TEM grids using a PMMA-assisted transfer method. Selected area electron diffraction (SAED), high-resolution transmission electron microscopy (HRTEM), energy-dispersive X-ray spectroscopy, high-angle annular dark-field scanning transmission electron microscopy (HAADF-STEM), and EDS mapping were obtained using a Thermo Fisher Talos F200X instrument at 120 kV acceleration voltage.

X-ray diffraction (XRD): Bruker D8 X-ray diffractometer with Cu-Kα radiation (λ = 1.54056 Å) operated at 40 kV and 40 mA.

### 2.4. Methods

#### 2.4.1. Preparation of Si/SiO_2_ Substrate

Prior to the growth, the Si/SiO_2_ substrates (1 cm × 1 cm) were cleaned in acetone, anhydrous ethanol, and deionized water by sonication for 15 min, respectively. The Si/SiO_2_ substrates were then dried by N_2_ (99.999%) and set aside for further processes.

#### 2.4.2. TEM Sample Preparation

The transfer of 2D materials involves challenges that can affect the quality and performance of the resulting layers. Polymer-assisted transfer techniques, where 2D materials are picked up from a substrate and released onto a target, can lead to contamination, mechanical stress-induced wrinkles, and cracks, degrading device performance [47,48]. Recent advancements have addressed these issues. Polymer-Assisted Transfer: Using a polyvinyl chloride-covered PDMS microdome with a van der Waals (vdW)-assisted layer prevents direct contact between the polymer and the 2D material, reducing contamination and enhancing interface quality for better device performance [49]. Se-Assisted Exfoliation: Selenium-assisted exfoliation allows for clean transfers with minimal defects, offering a scalable method for producing high-quality monolayer TMDCs [50]. The transfer methods used in this thesis are described below: firstly, the Si/SiO_2_ substrate grown with V-WSe_2_ was spin-coated with poly methyl methacrylate (PMMA), and then etched in NaOH solution, which reacts with SiO_2_ to remove the oxidized layer of the silicon substrate, so that the PMMA with samples is separated from the substrate, and the PMMA was washed in deionized water 3–5 times. After drying, the PMMA/V-WSe_2_ membrane was carefully lifted using a copper grid. Finally, the PMMA layer was dissolved with acetone, leaving the V-WSe_2_ sample on the copper grid.

## 3. Results and Discussion

**Figure 1:**
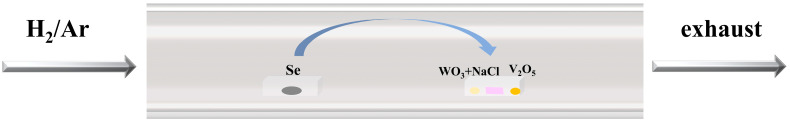
Schematic diagram of V-WSe_2_ synthesis by APCVD method.

Figure 1 illustrates the schematic diagram of the CVD synthesis process for V-WSe_2_. The metallic precursor powders were placed in a ceramic boat at the center of the heating zone in a tube furnace, while the silicon substrate was positioned between the tungsten oxide (WO_3_) and vanadium oxide (V_2_O_5_) powders. Selenium (Se) powder was placed separately in a ceramic boat located upstream. The entire growth process was conducted under the assistance of a carrier gas, which transported the precursor vapors to the substrate surface, where they reacted to form the target product, V-WSe_2_. To determine the optimal halide ratio, experiments were performed by varying the amount of NaCl under identical conditions. As shown in Figure S1, when the NaCl content was 5%, large-area monolayer V-WSe_2_ samples were successfully synthesized. Increasing the NaCl content to 10% resulted in the formation of thick island-like V-WSe_2_, along with the appearance of by-products and impurities on the substrate. Further increasing the NaCl content to 50% yielded bulk-like V-WSe_2_ and a higher quantity of by-products. This phenomenon is attributed to the rapid formation of the intermediate product WOCl_4_ during the reaction due to the excessive amount of NaCl, which accelerates the synthesis of V-WSe_2_ and leads to the formation of thick island-like structures. Based on these experimental results, a NaCl content of 5% was identified as the most suitable for the synthesis. Consequently, all subsequent orthogonal experiments were conducted using halide concentrations fixed at 5%. Additionally, the carrier gas flow rate and the distance between the substrate and the precursor powders were found to have significant effects on the synthesis of V-WSe_2_. The optimization of these two parameters is detailed in Supplementary Figures S2 and S3.

**Figure 2:**
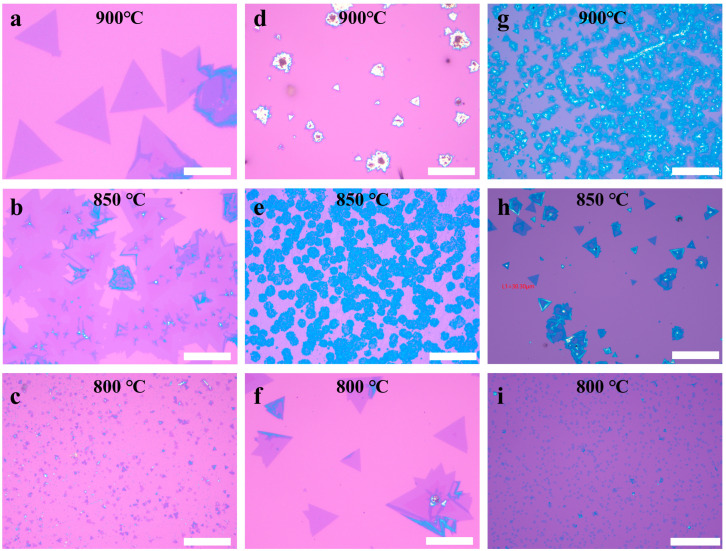
Synthesis of V-WSe_2_ using different halides at different growth temperatures: (**a**–**c**) KCl-assisted synthesis of V-WSe_2_, (**d**–**f**) KI-assisted synthesis of V-WSe_2_, (**g**–**i**) NaCl-assisted synthesis of V-WSe_2_ (scale bar: 50 µm).

The halide-assisted atmospheric-pressure chemical vapor deposition (APCVD) method for synthesizing V-WSe_2_ effectively reduces the required reaction temperature. To investigate the effects of different types of halide on the growth of V-WSe_2_, this study employed three halide salts—KCl, KI, and NaCl—to synthesize V-WSe_2_ at various growth temperatures. At lower temperatures, reduced reactant concentration and mobility impede 2D flake growth. In contrast, temperatures exceeding 1000 °C result in degradation of monolayer WSe_2_, which significantly impacts its photoluminescence properties [51]. Thus, the 800–900 °C growth window was selected as an optimal range, providing a balance between achieving high-quality monolayers and avoiding the aforementioned issues. Figure 2a–c depicts V-WSe_2_ samples synthesized using KCl at different growth temperatures. At a growth temperature of 900 °C, the WO_3_, V_2_O_5_, and KCl powder mixture absorbed sufficient thermal energy to generate WOCl_4_, WO_2_X_2_, and VOCl_3_. The vapor reacted with selenium, transported by the Ar/H_2_ carrier gas mixture, to nucleate and grow on the silicon substrate. Under these conditions, V-WSe_2_ samples with uniform morphology and size distribution were successfully synthesized as monolayer films. The specific reaction process is shown in Equations (1) and (2). When the reaction temperature was reduced to 850 °C, the higher local vapor pressure in the tube furnace, resulting from the increased concentration of reactants, led to island-like growth. The resulting samples were multilayered and exhibited a higher density of nucleation sites on their surface. Further reducing the reaction temperature to 800 °C resulted in V-WSe_2_ samples with very small lateral sizes. This was attributed to the insufficient vaporization of the precursors at low temperatures, as the limited amount of precursor vapor was only enough to support nucleation on the substrate, thereby restricting the growth size. Consequently, the products primarily consisted of triangular domains measuring only a few micrometers in size. When KI was used as the halide additive (Figure 2d–f), the reaction temperature could be reduced to as low as 800 °C, while still achieving effective growth of the V-WSe_2_ monolayers. This reduction in temperature is a significant advantage, as it allows for better control over the size and morphology of the resulting 2D material. The presence of KI likely facilitates the sublimation of the precursor materials at lower temperatures, enhancing the precursor transport and deposition process, which is crucial for the formation of monolayer films. In contrast, when the reaction temperature was raised above the optimal range, several undesirable effects were observed. As the temperature increased, the rate of precursor deposition became much faster, which led to the growth of thicker and more disordered samples. Under these high-temperature conditions, the morphology of the samples became less defined, and they often lacked a consistent shape. This behavior is indicative of an uncontrolled growth mechanism, where the material tends to form more bulk-like structures rather than thin monolayers. Furthermore, as the temperature continued to increase beyond this point, the growth process shifted from two-dimensional (2D) to three-dimensional (3D), resulting in the formation of bulk-like structures. Thus, maintaining a controlled temperature range is crucial to achieving uniform monolayer growth and avoiding the transition to unwanted 3D bulk-like material. If the temperature of the reaction is high, the sample becomes thicker and has no fixed shape. Further increasing the temperature resulted in bulk-like growth of the samples. In contrast, NaCl-assisted growth (Figure 2g–i) allowed for a reduction in the reaction temperature to 850 °C. The minimum growth temperatures for the various halides are presented in Table 1. The experimental results indicate that, among the three halides (KCl, KI, and NaCl), KI leads to the lowest reaction temperature, which facilitates the growth of V-WSe_2_ at 800 °C. The lower reaction temperature achievable with KI is attributed to the larger ionic radius and lower polarity of the halide anion, which facilitate its separation from the halide cation. This enhances its ability to react with tungsten oxide, forming reactive intermediates. Consequently, the growth of KI-assisted V-WSe_2_ can be achieved at lower temperatures compared to the other halides.

WO_3_ (s) + KCl (s) + V_2_O_5_→KVO_3_ (s) + WOCl_4_ (g) + WO_2_Cl_2_ (g) + WO_2_ (s) + W (s) + VOCl_3_ (g) (1)

VOCl_3_ (g) + WOCl_4_ (g) + WO_2_Cl_2_ (g) +Se (g) + H_2_ (g)→V-WSe_2_ (s) + HCl (g) + H_2_O (g) (2)

**Figure 3:**
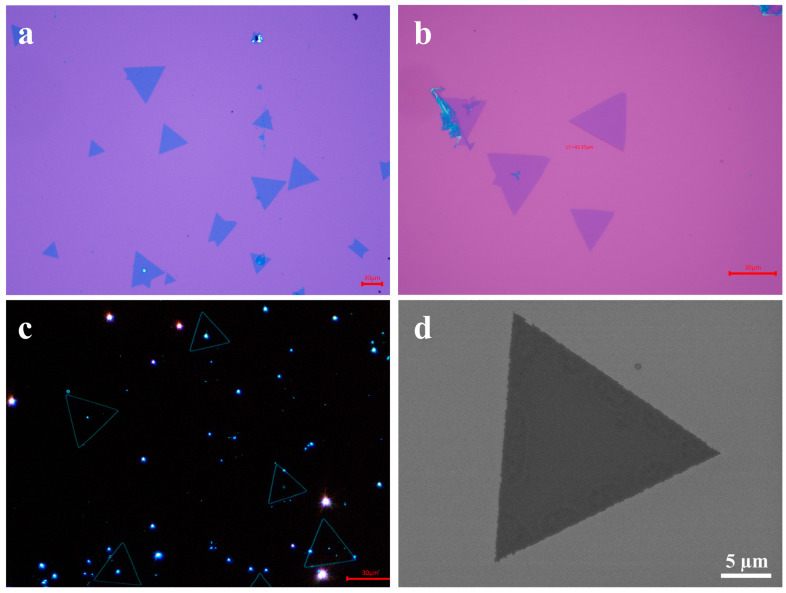
(**a**–**c**) Optical micrographs of V-WSe_2_; (**d**) scanning electron micrograph of V-WSe_2_.

To determine the morphology and crystal structure of the synthesized samples, optical microscopy (OM), field-emission scanning electron microscopy (SEM), and transmission electron microscopy (TEM) were used for characterization. Figure 3a shows a low-magnification optical microscopy image of monolayer V-WSe_2_, where the synthesized samples exhibit regular triangular morphologies. The size of the vanadium-doped WSe_2_ (V-WSe_2_) domains is approximately 30 µm, with sharp edges, uniform sizes, and an even distribution across the Si/SiO_2_ substrate. Figure 3b displays a high-magnification optical image, showing that the surface of the sample is clean, smooth, and nearly free of nucleation sites. Figure 3c presents a dark-field optical image, where the triangular-shaped samples exhibit green luminescence along their edges. The SEM image of monolayer V-WSe_2_ (Figure 3d) further confirms that the surface of the sample is clean and smooth, indicating a high crystalline quality of the synthesized material. To confirm the successful incorporation of vanadium into the WSe_2_ lattice, energy-dispersive X-ray spectroscopy (EDS) analysis was performed using SEM. The EDS spectrum clearly shows a distinct peak for vanadium (V), confirming the successful doping of vanadium into WSe_2_ (Figure S4).

**Figure 4:**
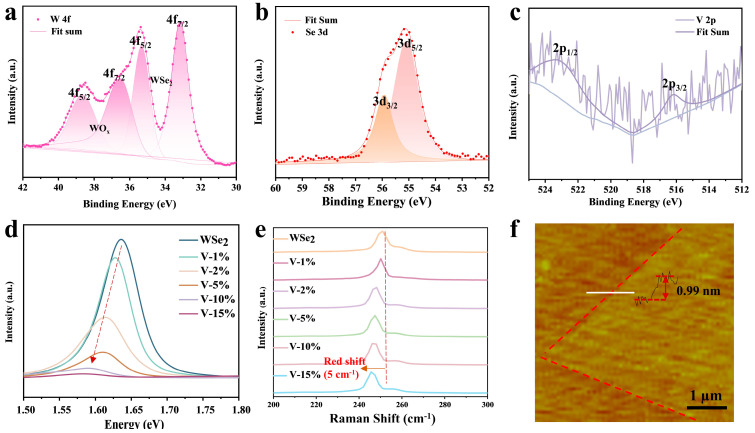
A series of characteristics of V-WSe_2_: (**a**) high-resolution XPS pattern of W in V-WSe_2_, (**b**) high-resolution XPS pattern of Se, (**c**) high-resolution XPS pattern of V, PL (**d**) and Raman (**e**) spectra of V-doped WSe_2_ monolayer in terms of V-doping concentration, and (**f**) atomic force microscopy images of V-WSe_2_.

Figure 4a–c shows the X-ray photoelectron spectroscopy (XPS) spectra of V-WSe_2_. Figure 4a presents the high-resolution XPS spectrum of W, where the peaks at 38.68 eV and 36.58 eV correspond to the W oxidation doublet. The presence of oxidation peaks is attributed to the relatively large analysis area (~500 × 500 µm^2^) of the XPS measurement. Since the CVD-synthesized V-WSe_2_ samples have domain sizes in the order of tens of micrometers, the measurement likely included contributions from the blank substrate. Additionally, during the high-temperature growth process, some tungsten oxide particles may have been deposited onto the substrate. The peaks at 35.28 eV and 33.18 eV correspond to the characteristic W 4f_5/2_ and 4f_7/2_ peaks, respectively [26,52,53,54]. In Figure 4b, the peaks at 55.95 eV and 55.08 eV correspond to the characteristic Se 3d_3/2_ and 3d_5/2_ peaks, respectively [55]. Figure 4c shows the high-resolution XPS spectrum of V, where the peaks at 523.18 eV and 516.18 eV correspond to the V^4+^ 2p_1/2_ and 2p_3/2_ characteristic peaks, respectively. These binding energies are consistent with previously reported values in the literature [56], confirming the successful incorporation of vanadium ions from the V_2_O_5_ precursor into the WSe_2_ lattice. Due to the very low doping concentration of vanadium, the signal intensity in the high-resolution XPS spectrum of V is relatively weak. The full XPS spectrum (Figure S5) further verifies the direct synthesis of V-WSe_2_ samples. To examine the influence of vanadium concentration on the properties of WSe_2_, we synthesized V-doped WSe_2_ at varying vanadium concentrations. As shown in Figure S6, increasing V doping concentration induces a distinct transformation in WSe_2_ morphology from triangular to near-hexagonal with jagged edges. This suggests that V incorporation disrupts the anisotropic growth dynamics of WSe_2_, likely due to lattice distortion and strain introduced by the V substituted W. Figure 4d,e presents the optical properties of V-WSe_2_, characterized by confocal photoluminescence (PL) spectroscopy and confocal Raman spectroscopy. PL spectra revealed a redshift in the emission peak of V-doped WSe_2_ compared to the undoped sample, indicating a reduction in bandgap. As the vanadium doping concentration increases, the photoluminescence (PL) intensity of WSe_2_ crystals gradually decreases. This aligns with the introduction of impurity states near the valence band edge due to V doping, which modifies the electronic structure. By adjusting the vanadium precursor ratio, we achieved a continuous modulation of the bandgap from 1.64 eV for pristine WSe_2_ to 1.58 eV with 15 wt% vanadium doping [55,57]. The Raman peak near 250 cm^−1^ is attributed to the mixed mode of E^1^_2g_ (in-plane vibrations of W and Se) and A_1g_ (out-of-plane vibrations of Se), while the 2LA(M) mode appears near 260 cm^−1^ [55,58]. The A_1g_ and E^1^_2g_ Raman modes in V-doped WSe_2_ exhibited a measurable redshift (~5 cm^−1^), suggesting lattice softening or strain effects induced by V substitution at W sites [59,60]. Figure S7 shows the Raman mapping, where the Raman intensity distribution within the selected area is uniform, indicating that the monolayer V-WSe_2_ material exhibits high crystallinity and low defect density. The atomic force microscopy (AFM) image of V-WSe_2_ (Figure 4f) reveals sharp sample edges and a smooth surface, with no visible cracks. The sample exhibits uniform contrast across the entire flake. Height profiling along the white line in the image shows a thickness of 0.99 nm, confirming that the synthesized V-WSe_2_ is monolayer [51].

**Figure 5:**
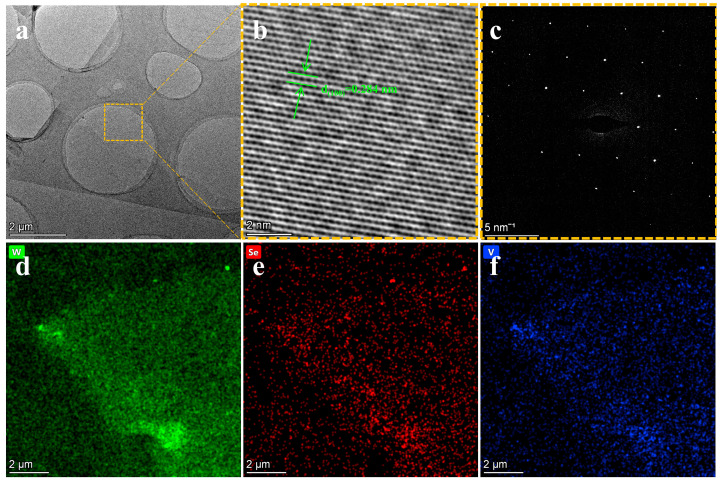
Structure and element analysis of V-WSe_2_: (**a**) TEM image of V-WSe_2_ at low magnification, (**b**) HRTEM image of the boxed region in (**a**), (**c**) selected electron diffraction of the boxed region in (**a**), and (**d**–**f**) EDS-mapping images of V-WSe_2_.

To further investigate the microstructure of V-WSe_2_, high-resolution transmission electron microscopy (HRTEM) analysis was conducted (Figure 5). Since TEM observations require samples to be mounted on copper grids, the V-WSe_2_ samples grown on Si/SiO_2_ substrates were transferred onto 200-mesh copper grids with standard microgrid support films. The detailed sample transfer procedure is described in the Methods section. Figure 5a shows a low-magnification TEM image of V-WSe_2_. A higher-magnification analysis of the region outlined in Figure 5a is shown in Figure 5b, where the lattice spacing of 0.284 nm corresponds to the (100) crystallographic plane of WSe_2_ [61]. No significant changes in the lattice parameters were observed, indicating that vanadium doping occurred via substitutional doping. Figure 5c presents the selected area electron diffraction (SAED) pattern of V-WSe_2_, with the zone axis along [001]. The bright diffraction spots correspond to the (100) planes [61], and the diffraction pattern is consistent with the atomic structure of WSe_2_. This result confirms that the V-WSe_2_ synthesized via chemical vapor deposition exhibits high crystalline quality, and the lattice parameters of tungsten diselenide remain nearly unchanged after vanadium doping. Figure 5d–f displays the EDS mapping images of V-WSe_2_ obtained in HAADF-STEM mode, showing the distribution of W (green), Se (red), and V (blue). The mapping results indicate a relatively uniform distribution of all three elements within the V-WSe_2_ sample. Furthermore, to confirm that the V-WSe_2_ samples exhibit a [001] crystallographic orientation, X-ray diffraction (XRD) analysis was performed on the Si/SiO_2_ substrate with monolayer V-WSe_2_ (Figure S8). The XRD pattern displays diffraction peaks corresponding to the (002), (006), and (008) planes, indicating that the synthesized V-WSe_2_ has a layered structure [62,63].

## 4. Conclusions

In summary, we successfully synthesized V-WSe_2_ with varying V concentrations (1%, 2%, 5%, 10%, 15%) using a halide-assisted APCVD method. Three halide salts—KCl, NaCl, and KI—were employed to facilitate growth at different temperatures. With the assistance of KCl, the growth temperature could be reduced to 900 °C, while NaCl reduced the reaction temperature to 850 °C. KI enabled the lowest growth temperature of 800 °C, compared to the growth temperature range of 950–1000 °C without halide assistance. The superior performance of KI in reducing the reaction temperature is attributed to its ability to readily react with tungsten oxide to form intermediate products, thereby lowering the energy barrier for the volatilization of tungsten oxide. Comprehensive characterization of the synthesized samples demonstrated that the V-WSe_2_ exhibits high crystalline quality, well-defined morphology, uniform size distribution, and smooth, uncontaminated surfaces. Furthermore, by varying the vanadium ratio, we achieved a continuous modulation of the bandgap from 1.64 eV for pristine WSe_2_ to 1.58 eV with 15 wt% vanadium doping and Raman modes in V-doped WSe_2_ exhibited a measurable redshift (~5 cm^−1^). SEM-EDS, STEM-EDS, and XPS analyses confirmed the successful incorporation of vanadium atoms into the WSe_2_ lattice, verifying the successful synthesis of V-WSe_2_.

## Figures and Tables

**Table 1:** Minimum growth temperature for halide-assisted synthesis of V-WSe_2_.

Halide	KCl	KI	NaCl
Temperature (°C)	900	800	850

## Data Availability

The original contributions presented in this study are included in the article/Supplementary Materials. Further inquiries can be directed to the corresponding author.

## Supplementary Materials

The following supporting information can be downloaded at: https://www.mdpi.com/article/10.3390/ma18235360/s1, Figure S1. Optical micrographs of V-WSe_2_ prepared with different NaCl contents; Figure S2. Optical micrographs of V-WSe_2_ prepared with different carrier gas flow rate; Figure S3. Optical micrographs of V-WSe_2_ prepared at different WO_3_ precursor-substrate distances; Figure S4. SEM images and SEM-EDS of V-WSe_2_; Figure S5. Full XPS spectrum of V-WSe_2_; Figure S6. Optical micrographs of V-WSe_2_ at different V doping concentration. (**a**) V-2%, (**b**) V-5%, (**c**) V-10% mm, (**d**) V-15%; Figure S7. Raman mapping (**a**) Optical micrographs of V-WSe_2_, (**b**) Raman intensity distribution in the selected region in (**a**); Figure S8. XRD analysis of V-WSe_2_.

## References

[1] Novoselov K.S., Geim A.K., Morozov S.V., Jiang D., Zhang Y., Dubonos S.V., Grigorieva I.V., Firsov A.A. (2004). Electric field effect in atomically thin carbon films. Science.

[2] Novoselov K.S., Fal’ko V.I., Colombo L., Gellert P.R., Schwab M.G., Kim K. (2012). A roadmap for graphene. Nature.

[3] Grigorenko A.N., Polini M., Novoselov K.S. (2012). Graphene plasmonics. Nat. Photonics.

[4] Balandin A.A., Ghosh S., Bao W.Z., Calizo I., Teweldebrhan D., Miao F., Lau C.N. (2008). Superior thermal conductivity of single-layer graphene. Nano Lett..

[5] Yin Y., Kang X., Han B. (2022). Two-dimensional materials: Synthesis and applications in the electro-reduction of carbon dioxide. Chem. Synth..

[6] Sakurai M. (2014). On-surface synthesized magnetic nanoclusters of ferrocene derivatives. Chem. Synth..

[7] Tang Z., Zhao D., Wang X., Jiao Y., Liu M., Liu C., Zhang Q., Ren S., Liu Y. (2025). One-Pot Synthesis of Pd@Pt Core-Shell Icosahedron for Efficient Oxygen Reduction. Materials.

[8] Chhowalla M., Shin H.S., Eda G., Li L.J., Loh K.P., Zhang H. (2013). The chemistry of two-dimensional layered transition metal dichalcogenide nanosheets. Nat. Chem..

[9] Zhou J.D., Zhu C., Zhou Y., Dong J.C., Li P.L., Zhang Z.W., Wang Z., Lin Y.C., Shi J., Zhang R.W. (2023). Composition and phase engineering of metal chalcogenides and phosphorous chalcogenides. Nat. Mater..

[10] Manzeli S., Ovchinnikov D., Pasquier D., Yazyev O.V., Kis A. (2017). 2D transition metal dichalcogenides. Nat. Rev. Mater..

[11] Mak K.F., Lee C., Hone J., Shan J., Heinz T.F. (2010). Atomically Thin MoS_2_: A New Direct-Gap Semiconductor. Phys. Rev. Lett..

[12] Splendiani A., Sun L., Zhang Y.B., Li T.S., Kim J., Chim C.Y., Galli G., Wang F. (2010). Emerging Photoluminescence in Monolayer MoS_2_. Nano Lett..

[13] Lembke D., Bertolazzi S., Kis A. (2015). Single-Layer MoS_2_ Electronics. Acc. Chem. Res..

[14] Hwangbo S., Hu L., Hoang A.T., Choi J.Y., Ahn J.H. (2022). Wafer-scale monolithic integration of full-colour micro-LED display using MoS_2_ transistor. Nat. Nanotechnol..

[15] Wang Q.H., Kalantar-Zadeh K., Kis A., Coleman J.N., Strano M.S. (2012). Electronics and optoelectronics of two-dimensional transition metal dichalcogenides. Nat. Nanotechnol..

[16] Wu S.F., Buckley S., Schaibley J.R., Feng L.F., Yan J.Q., Mandrus D.G., Hatami F., Yao W., Vuckovic J., Majumdar A. (2015). Monolayer semiconductor nanocavity lasers with ultralow thresholds. Nature.

[17] Choi M., Bae S.R., Hu L., Hoang A.T., Kim S.Y., Ahn J.H. (2020). Full-color active-matrix organic light-emitting diode display on human skin based on a large-area MoS_2_ backplane. Sci. Adv..

[18] Hoang A.T., Hu L.H., Kim B.J., Van T.T.N., Park K.D., Jeong Y., Lee K., Ji S., Hong J., Katiyar A.K. (2023). Low-temperature growth of MoS_2_ on polymer and thin glass substrates for flexible electronics. Nat. Nanotechnol..

[19] Wang S.Y., Liu X.X., Xu M.S., Liu L.W., Yang D.R., Zhou P. (2022). Two-dimensional devices and integration towards the silicon lines. Nat. Mater..

[20] Wang S.Y., Liu X.X., Zhou P. (2022). The Road for 2D Semiconductors in the Silicon Age. Adv. Mater..

[21] Ahn E.C. (2020). 2D materials for spintronic devices. npj 2D Mater. Appl..

[22] Ross J.S., Rivera P., Schaibley J., Lee-Wong E., Yu H.Y., Taniguchi T., Watanabe K., Yan J.Q., Mandrus D., Cobden D. (2017). Interlayer Exciton Optoelectronics in a 2D Heterostructure p-n Junction. Nano Lett..

[23] Liu L.T., Kumar S.B., Ouyang Y., Guo J. (2011). Performance Limits of Monolayer Transition Metal Dichalcogenide Transistors. IEEE Trans. Electron Devices.

[24] Feng L.P., Jiang W.Z., Su J., Zhou L.Q., Liu Z.T. (2016). Performance of field-effect transistors based on Nb_x_W_1-x_S_2_ monolayers. Nanoscale.

[25] Walia S., Balendhran S., Wang Y.C., Ab Kadir R., Zoolfakar A.S., Atkin P., Ou J.Z., Sriram S., Kalantar-zadeh K., Bhaskaran M. (2013). Characterization of metal contacts for two-dimensional MoS_2_ nanoflakes. Appl. Phys. Lett..

[26] Das S., Chen H.Y., Penumatcha A.V., Appenzeller J. (2013). High Performance Multilayer MoS_2_ Transistors with Scandium Contacts. Nano Lett..

[27] Radisavljevic B., Radenovic A., Brivio J., Giacometti V., Kis A. (2011). Single-layer MoS_2_ transistors. Nat. Nanotechnol..

[28] Kozhakhmetov A., Stolz S., Tan A.M.Z., Pendurthi R., Bachu S., Turker F., Alem N., Kachian J., Das S., Hennig R.G. (2021). Controllable p-Type Doping of 2D WSe_2_ via Vanadium Substitution. Adv. Funct. Mater..

[29] Xu H.L., Fathipour S., Kinder E.W., Seabaugh A.C., Fullerton-Shirey S.K. (2015). Reconfigurable Ion Gating of 2H-MoTe_2_ Field-Effect Transistors Using Poly(ethylene oxide)-CsClO_4_ Solid Polymer Electrolyte. ACS Nano.

[30] Zhang K.H., Feng S.M., Wang J.J., Azcatl A., Lu N., Addou R., Wang N., Zhou C.J., Lerach J., Bojan V. (2016). Manganese Doping of Monolayer MoS_2_: The Substrate Is Critical. Nano Lett..

[31] Mouri S., Miyauchi Y., Matsuda K. (2013). Tunable Photoluminescence of Monolayer MoS_2_ via Chemical Doping. Nano Lett..

[32] Fang H., Tosun M., Seol G., Chang T.C., Takei K., Guo J., Javey A. (2013). Degenerate n-Doping of Few-Layer Transition Metal Dichalcogenides by Potassium. Nano Lett..

[33] Ryu M.Y., Jang H.K., Lee K.J., Piao M.X., Ko S.P., Shin M., Huh J., Kim G.T. (2017). Triethanolamine doped multilayer MoS_2_ field effect transistors. Phys. Chem. Chem. Phys..

[34] Afaneh T., Sahoo P.K., Nobrega I.A.P., Xin Y., Gutiérrez H.R. (2018). Laser-Assisted Chemical Modification of Monolayer Transition Metal Dichalcogenides. Adv. Funct. Mater..

[35] Luo P., Zhuge F.W., Zhang Q.F., Chen Y.Q., Lv L., Huang Y., Li H.Q., Zhai T.Y. (2019). Doping engineering and functionalization of two-dimensional metal chalcogenides. Nanoscale Horiz..

[36] Fan M., Guo J., Fang G., Tian H., You Y., Huang Z., Huang J. (2024). Microwave-pulse assisted synthesis of tunable ternary-doped 2D molybdenum carbide for efficient hydrogen evolution. Chem. Synth..

[37] Zhao P.D., Kiriya D., Azcatl A., Zhang C.X., Tosun M., Liu Y.S., Hettick M., Kang J.S., McDonnell S., Santosh K.C. (2014). Air Stable p-Doping of WSe_2_ by Covalent Functionalization. ACS Nano.

[38] Wang S.F., Zhao W.J., Giustiniano F., Eda G. (2016). Effect of oxygen and ozone on p-type doping of ultra-thin WSe_2_ and MoSe_2_ field effect transistors. Phys. Chem. Chem. Phys..

[39] Das S., Demarteau M., Roelofs A. (2015). Nb-doped single crystalline MoS_2_ field effect transistor. Appl. Phys. Lett..

[40] Kozhakhmetov A., Schuler B., Tan A.M.Z., Cochrane K.A., Nasr J.R., El-Sherif H., Bansal A., Vera A., Bojan V., Redwing J.M. (2020). Scalable Substitutional Re-Doping and its Impact on the Optical and Electronic Properties of Tungsten Diselenide. Adv. Mater..

[41] Sun L.Z., Yuan G.W., Gao L.B., Yang J., Chhowalla M., Gharahcheshmeh M.H., Gleason K.K., Choi Y.S., Hong B.H., Liu Z.F. (2021). Chemical vapour deposition. Nat. Rev. Methods Primers.

[42] Hou T.Y., Li D., Qu Y., Hao Y.F., Lai Y. (2023). The Role of Carbon in Metal-Organic Chemical Vapor Deposition-Grown MoS_2_ Films. Materials.

[43] Luo X.M., Jiao Y.H., Li H., Liu Q., Liu J.F., Wang M.W., Liu Y. (2024). Impact of Carrier Gas Flow Rate on the Synthesis of Monolayer WSe_2_ via Hydrogen-Assisted Chemical Vapor Deposition. Materials.

[44] Gharahcheshmeh M.H., Gleason K.K. (2019). Device Fabrication Based on Oxidative Chemical Vapor Deposition (oCVD) Synthesis of Conducting Polymers and Related Conjugated Organic Materials. Adv. Mater. Interfaces.

[45] Heydari Gharahcheshmeh M. (2025). Fabrication of Conjugated Conducting Polymers by Chemical Vapor Deposition (CVD) Method. Nanomaterials.

[46] Li S., Wang S., Tang D.M., Zhao W., Xu H., Chu L., Eda G. (2015). Halide-assisted atmospheric pressure growth of large WSe_2_ and WS_2_ monolayer crystals. Appl. Mater. Today.

[47] Wang W.D., Clark N., Hamer M., Carl A., Tovari E., Sullivan-Allsop S., Tillotson E., Gao Y.Z., de Latour H., Selles F. (2023). Clean assembly of van der Waals heterostructures using silicon nitride membranes. Nat. Electron..

[48] Pham P.V., Mai T.H., Dash S.P., Biju V., Chueh Y.L., Jariwala D., Tung V. (2024). Transfer of 2D Films: From Imperfection to Perfection. ACS Nano.

[49] Wen S.F., Zhou S.R., Chen H.H., Gong Y.M., Kong L.K., Yin Y., Lan C.Y., Li C., Liu Y. (2025). Contamination-free assembly of two-dimensional van der Waals heterostructures toward high-performance electronics and optoelectronics. Appl. Mater. Today.

[50] Younas R., Zhou G.Y., Hinkle C.L. (2025). Scalable and Contamination-Free Selenium-Assisted Exfoliation of Transition Metal Dichalcogenides WSe_2_ and MoSe_2_. Processes.

[51] Liu B.L., Fathi M., Chen L., Abbas A., Ma Y.Q., Zhou C.W. (2015). Chemical Vapor Deposition Growth of Monolayer WSe_2_ with Tunable Device Characteristics and Growth Mechanism Study. ACS Nano.

[52] Chen J.Y., Liu B., Liu Y.P., Tang W., Nai C.T., Li L.J., Zheng J., Gao L.B., Zheng Y., Shin H.S. (2015). Chemical Vapor Deposition of Large-Sized Hexagonal WSe_2_ Crystals on Dielectric Substrates. Adv. Mater..

[53] Wang X.L., Gong Y.J., Shi G., Chow W.L., Keyshar K., Ye G.L., Vajtai R., Lou J., Liu Z., Ringe E. (2014). Chemical Vapor Deposition Growth of Crystalline Mono layer MoSe_2_. ACS Nano.

[54] Susarla S., Kutana A., Hachtel J.A., Kochat V., Apte A., Vajtai R., Idrobo J.C., Yakobson B.I., Tiwary C.S., Ajayan P.M. (2017). Quaternary 2D Transition Metal Dichalcogenides (TMDs) with Tunable Bandgap. Adv. Mater..

[55] Ji H.G., Solís-Fernández P., Yoshimura D., Maruyama M., Endo T., Miyata Y., Okada S., Ago H. (2019). Chemically Tuned p- and n-Type WSe_2_ Monolayers with High Carrier Mobility for Advanced Electronics. Adv. Mater..

[56] Yun S.J., Duong D.L., Ha D.M., Singh K., Phan T.L., Choi W., Kim Y.M., Lee Y.H. (2020). Ferromagnetic Order at Room Temperature in Monolayer WSe_2_ Semiconductor via Vanadium Dopant. Adv. Sci..

[57] Tonndorf P., Schmidt R., Böttger P., Zhang X., Börner J., Liebig A., Albrecht M., Kloc C., Gordan O., Zahn D.R.T. (2013). Photoluminescence emission and Raman response of monolayer MoS_2_, MoSe_2_, and WSe_2_. Opt. Express.

[58] Kang D.H., Shim J., Jang S.K., Jeon J., Jeon M.H., Yeom G.Y., Jung W.S., Jang Y.H., Lee S., Park J.H. (2015). Controllable Nondegenerate p-Type Doping of Tungsten Diselenide by Octadecyltrichlorosilane. ACS Nano.

[59] Wu K., Wang H., Yang M., Liu L., Sun Z.Y., Hu G.J., Song Y.P., Han X., Guo J.A., Wu K.H. (2024). Gold-Template-Assisted Mechanical Exfoliation of Large-Area 2D Layers Enables Efficient and Precise Construction of Moiré Superlattices. Adv. Mater..

[60] Faggio G., Politano G.G., Lisi N., Capasso A., Messina G. (2024). The structure of chemical vapor deposited graphene substrates for graphene-enhanced Raman spectroscopy. J. Phys. Condens. Matter.

[61] Duan X.D., Wang C., Shaw J.C., Cheng R., Chen Y., Li H.L., Wu X.P., Tang Y., Zhang Q.L., Pan A.L. (2014). Lateral epitaxial growth of two-dimensional layered semiconductor heterojunctions. Nat. Nanotechnol..

[62] Kim H., Yun S.J., Park J.C., Park M.H., Park J.H., Kim K.K., Lee Y.H. (2015). Seed Growth of Tungsten Diselenide Nanotubes from Tungsten Oxides. Small.

[63] Zhang Y.X., Wang Y.H., Xiong Z.Z., Zhang H.J., Liang F. (2018). Preparation and characterization of WSe_2_ nano-films by magnetron sputtering and vacuum selenization. Nanotechnology.

