# Supplementary material for: Halide-Assisted Synthesis of V-WSe2
Source: Materials (Basel). 2025 Nov 28;18(23):5360. doi: 10.3390/ma18235360 (PMC12693213; doi:10.3390/ma18235360)
Supplement: Supplementary file 1 [file materials-18-05360-s001.zip › materials-3527607-supplementary.pdf]

Supporting Information

Halide-assisted synthesis of V-WSe_2

Yanhui Jiao, Xiaoqian Wang, Zisheng Tang, Manrui Liu, Chenqi Liu, Qi Zhang, and Yong Liu *

State Key Laboratory of Advanced Technology for Materials Synthesis and Processing, International School of Materials Science and Engineering (ISMSE), Wuhan University of Technology, Wuhan 430070, China; j1769877205@163.com (Y.J.); wangxiaoqian0@163.com (X.W.); tangzs3076@163.com (Z.T.); liumanr14@163.com (M. L.); liuchengqi42@163.com (C.L.); zq13307239180@163.com (Q.Z.).

* Correspondence: liuyong3873@whut.edu.cn

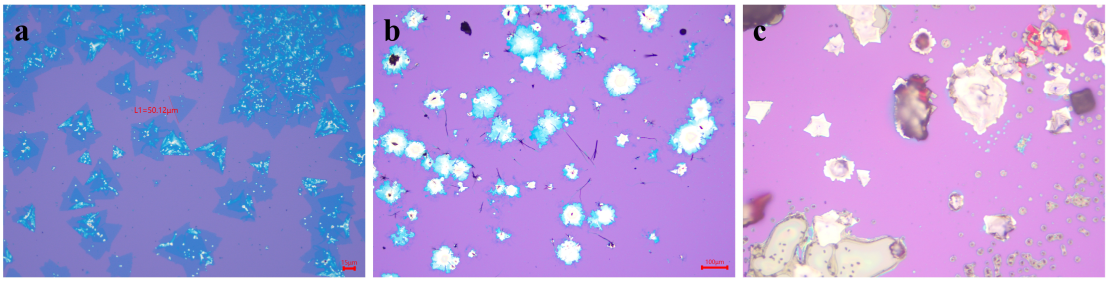

Figure S1 Optical micrographs of V-WSe_2 prepared with different NaCl contents (**a**) 5%, (**b**) 10%, (**c**) 50%.

During the chemical vapor deposition (CVD) synthesis of V-WSe_2, hydrogen gas is required to assist the growth process due to the weaker reducing ability of selenium powder compared to sulfur powder. For safety considerations, a hydrogen-argon mixture containing 10% hydrogen was used as the carrier gas. The flow rate of the carrier gas significantly affects the synthesis of V-WSe_2, particularly under high-temperature growth conditions. To optimize the process, we investigated the growth of V-WSe_2 under various carrier gas flow rates.

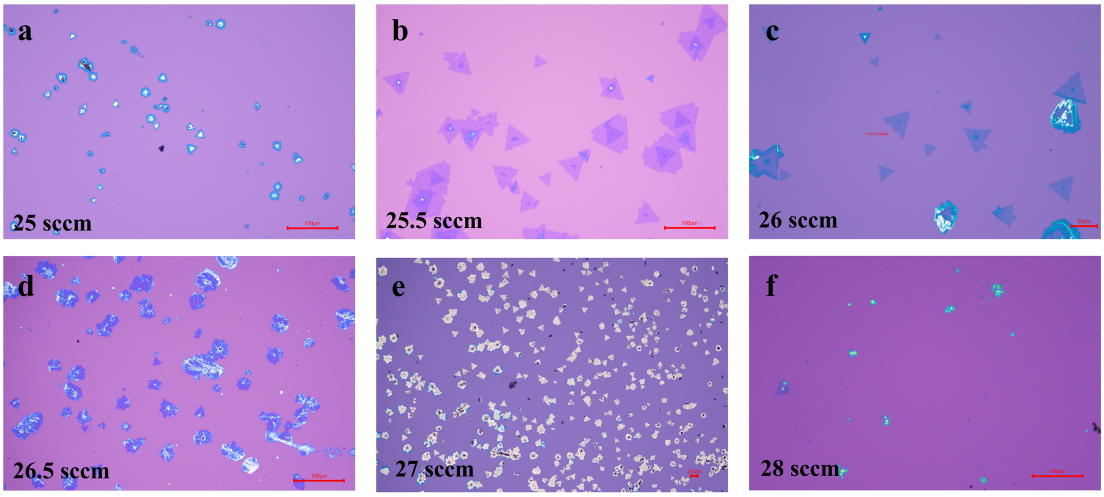

Figure S2 Optical micrographs of V-WSe_2 prepared with different carrier gas flow rate. (**a**) 25 sccm, (**b**) 25.5 sccm, (**c**) 26 sccm, (**d**) 26.5 sccm, (**e**) 27 sccm, (**f**) 28 sccm.

Figures S2 a–f shows the optical microscopy images of V-WSe_2 samples synthesized at carrier gas flow rates ranging from 25 sccm to 28 sccm. At low flow rates, the carrier gas was insufficient to transport an adequate amount of precursor to the silicon substrate, resulting in sparse and small-sized samples on the substrate. Increasing the carrier gas flow rate to an optimal level facilitated the transport of an appropriate amount of precursor to the substrate, enabling the growth of monolayer samples with well-defined morphology. However, further increasing the carrier gas flow rate caused the edges of the samples to exhibit a curled morphology, likely due to the higher flow rate lifting the sample edges during lateral growth, leading to locally thicker regions. When the flow rate was increased further, an excessive amount of precursor was delivered to the substrate within a short period, resulting in a high nucleation density and the growth of thicker samples, approaching bulk-like structures. At excessively high flow rates, the carrier gas swept the precursor past the substrate, leaving almost no material deposited on the

substrate surface. Based on the analysis of the experimental results, the most suitable carrier gas flow rates were determined to be 25.5 sccm and 26 sccm. However, at 25.5 sccm, the samples were mostly bilayered with noticeable nucleation points on the surface. Therefore, a flow rate of 26 sccm was selected for subsequent experiments to achieve optimal sample quality.

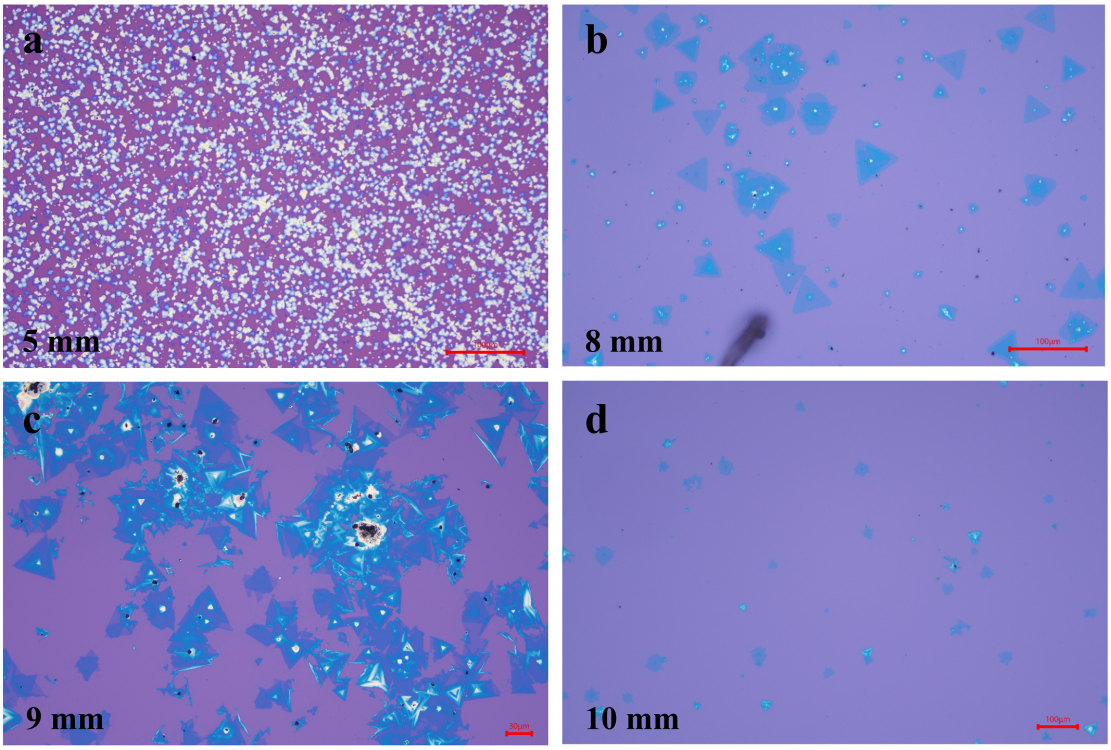

Figure S3 Optical micrographs of V-WSe_2 prepared at different WO_3 precursor-substrate distances. (**a**) 5 mm, (**b**) 8 mm, (**c**) 9 mm, (**d**) 10 mm.

In addition to the carrier gas flow rate and growth temperature, the distance between the precursor and the substrate also plays a critical role in the growth of V-WSe_2. To determine the optimal distance, the effect of varying the distance between the tungsten oxide (WO_3) precursor and the silicon substrate was investigated. Figure S3 shows optical microscopy images of V-WSe_2 samples synthesized at different precursor-to-substrate distances. When the precursor was placed too close to the substrate, the nucleation density on the silicon substrate was excessively high, resulting in small-sized, thick samples. Conversely, when the precursor was positioned too far from the substrate, the carrier gas could not effectively transport sufficient precursor to the substrate for the reaction, leading to sparse and irregularly shaped samples. Based on these observations, the optimal precursor-to-substrate distance was determined to be approximately 8 mm.

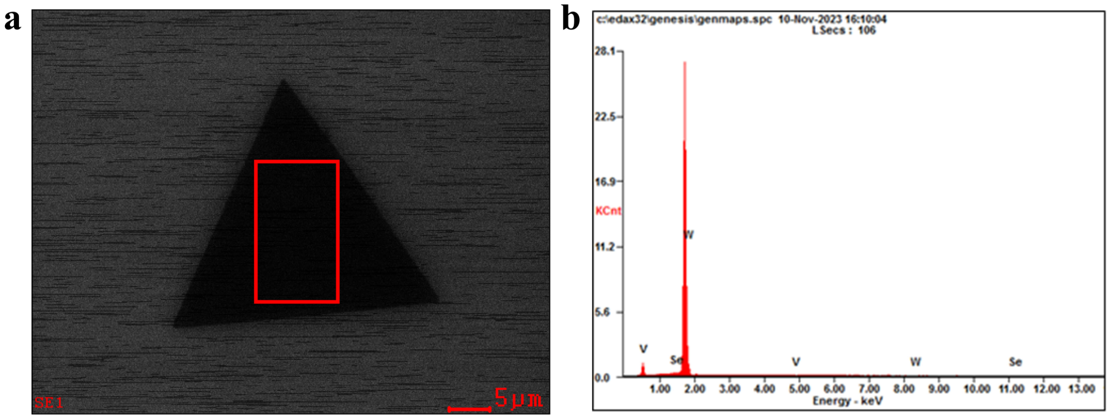

Figure S4 (**a**) SEM of V-WSe_2, (**b**) SEM-EDS of V-WSe_2.

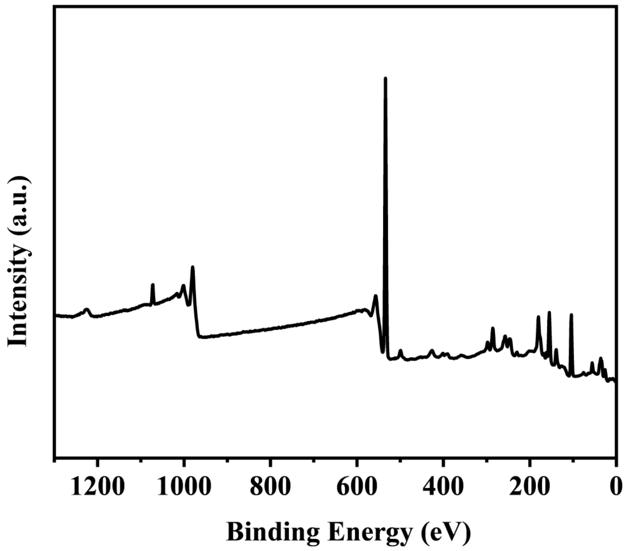

Figure S5 Full XPS spectrum of V-WSe_2.

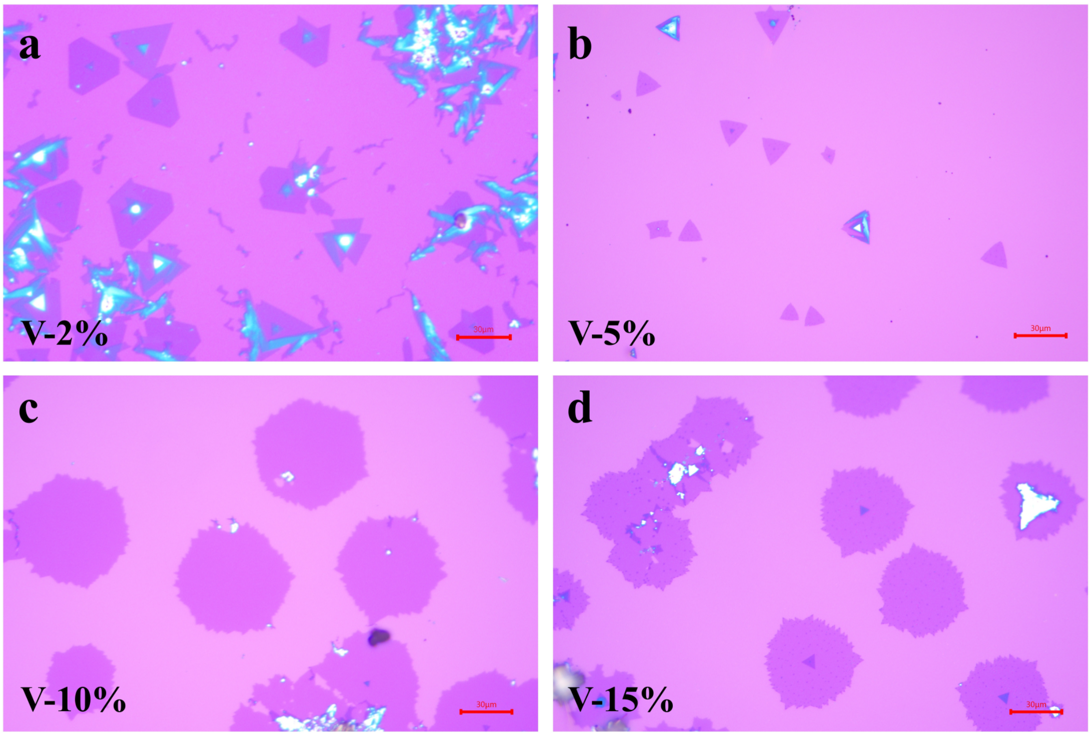

Figure S6 Optical micrographs of V-WSe_2 at different V doping concentration. (**a**) V-2%, (**b**) V-5%, (**c**) V-10% mm, (**d**) V-15%.

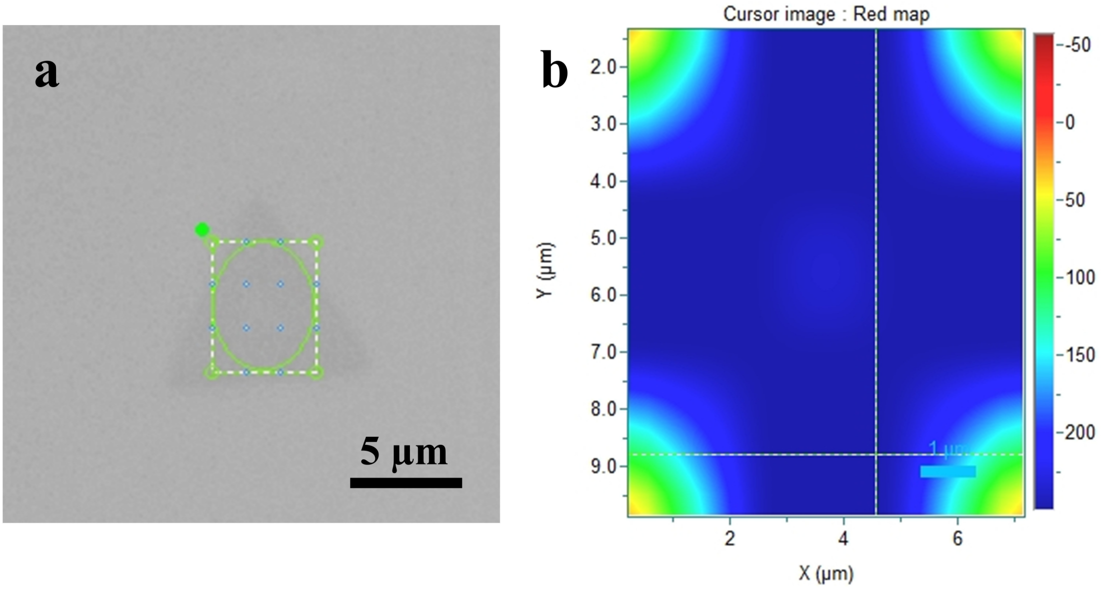

Figure S7 Raman mapping (**a)** Optical micrographs of V-WSe_2, (**b**) Raman intensity distribution in the selected region in (a).

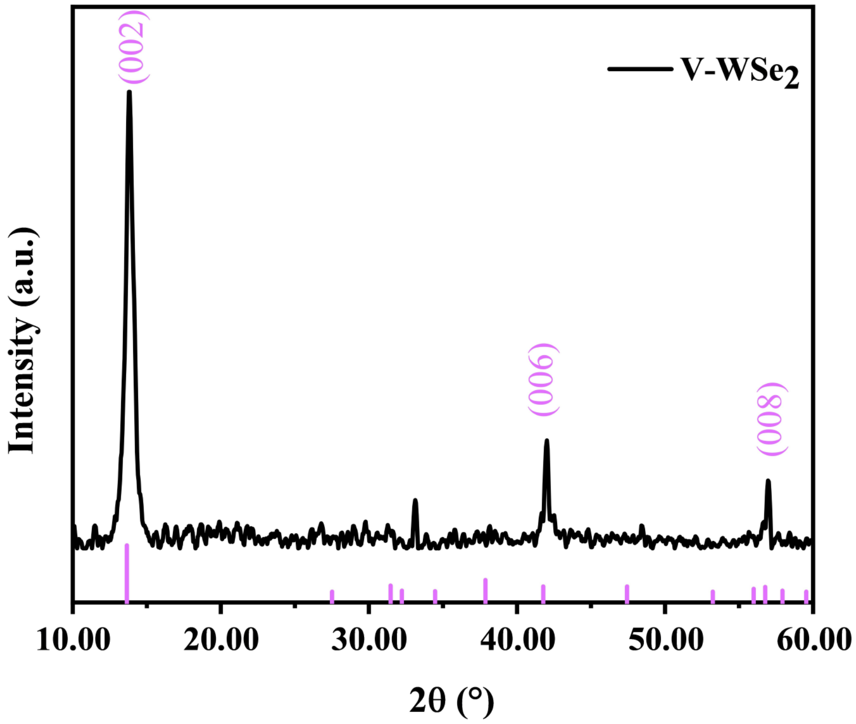

Figure S8 XRD analysis of V-WSe_2.
